# A qualitative exploration of ambulance clinician behaviour and decision making to identify factors influencing on-scene times for suspected stroke patients in North East England

**DOI:** 10.29045/14784726.2024.3.8.4.1

**Published:** 2024-03-01

**Authors:** Abi Alton, Lisa Shaw, Tracy Finch, Christopher Price, Graham McClelland

**Affiliations:** Newcastle University ORCID iD: https://orcid.org/0000-0002-9983-080X; Newcastle University ORCID iD: https://orcid.org/0000-0002-3435-9519; Northumbria University ORCID iD: https://orcid.org/0000-0001-8647-735X; Newcastle University ORCID iD: https://orcid.org/0000-0003-3566-3157; North East Ambulance Service NHS Foundation Trust ORCID iD: https://orcid.org/0000-0002-4502-5821

**Keywords:** on-scene time, pre-hospital, stroke

## Abstract

**Aims/objectives:**

Ambulance clinician assessment of suspected stroke patients aims to provide rapid access to specialist care, however regional and national data show increasing pre-hospital times. This study explored paramedic views about factors contributing to on-scene time (OST) for suspected stroke patients, with a view to identifying opportunities for future interventions, to reduce OST.

**Methods:**

Views of paramedics from one regional service on factors influencing OST were explored using a qualitative approach. Semi-structured interviews with volunteers were recorded, transcribed and analysed using thematic analysis.

**Results:**

Interviews were conducted with 13 paramedics between August and November 2021. Five interlinked themes were identified and described a range of factors influencing OST:

1. ‘Initial assessment and sources of information’ describes how clinicians make assessments based on initial presentation, influenced by pre-arrival information from ambulance control and family members / bystanders at the scene, and how this influences OST.
2. ‘Suitability for treatment and interventions’ describes how paramedics consider actions such as the face, arms, speech test, cannulation, electrocardiograms and neurological assessments while recognising that pre-hospital interventions for suspected stroke are limited.
3. ‘The environment’ describes the influence of incident setting on OST, including the overall process needed to transport the patient to appropriate care.
4. ‘Hospital interactions’ describes how interactions with hospital staff influenced paramedic actions and OST.
5. ‘Changing practice’ describes the influence of experience and interaction with hospital staff leading to changes in paramedic practice over time.

**Conclusion:**

This study provides insight into how UK paramedics spend time on scene with stroke patients. Multiple factors influencing OST were identified which signpost opportunities for interventions designed to reduce OST. Standardising on-scene assessments for stroke patients, refining communication processes between ambulance services and hospital stroke services and increasing availability of stroke continuing professional development for paramedics were all identified as potential targets for improving OST.

## Introduction

Rapid access to specialist units is vital for stroke patients, due to the time-dependent nature of emergency treatments, and evidence that better outcomes result from preventing early complications through standardisation of care (Fulop et al., 2019; Langhorne et al., 2020). In particular, thrombolysis and thrombectomy for ischaemic stroke treatment are most effective when administered soon after stroke onset, with standard treatment windows of 4.5 and 6 hours respectively (Evans et al., 2017; Wardlaw et al., 2014).

Around two-thirds of acute stroke patients in England are taken to hospital by emergency ambulance (Price et al., 2013). Because of time-critical circumstances, pre-hospital stroke care aims to achieve prompt recognition of stroke symptoms followed by rapid transport to specialist care. Despite these aims, evidence suggests time to hospital arrival is increasing for patients with stroke symptoms. Data from the North East Ambulance Service NHS Foundation Trust (NEAS) showed that pre-hospital call-to-hospital times for stroke patients increased from 41 to 68 minutes between 2011 and 2018 (Haworth & McClelland, 2019). A recent Sentinel Stroke National Audit Programme report also highlighted that across England, Wales and Northern Ireland, call-to-hospital times increased by nine minutes between April 2020 and January 2021 (Sentinel Stroke National Audit Programme [SSNAP], 2020). NHS stroke care recommendations (Getting It Right First Time, 2022) continue to highlight the need to improve the pre-hospital stroke pathway, with a view to reducing delays in accessing specialist care.

The pre-hospital stroke care pathway comprises four phases: (1) symptom onset to call for help; (2) call for help to arrival of help; (3) on-scene time (OST); and (4) travel to hospital. OST may be most amenable to efficiency improvements as it mostly reflects clinician activity. UK ambulance services report an average OST of 30–31 minutes (Haworth & McClelland, 2019; London Ambulance Service NHS Trust, 2018). However, other countries report shorter OSTs, with a median of 15 minutes in the United States (Schwartz et al., 2016), and mean 23 minutes in Australia (Fouche et al., 2019). Danish services report 18 minutes OST, which accounts for 44% of the call-to-hospital time (Simonsen et al., 2014). Although these times reflect contextual factors such as local stroke assessment guidelines, they suggest that there may be an opportunity to reduce UK OST. This may be achieved through training and setting OST targets (Puolakka et al., 2016); however, without understanding the factors affecting OST for UK ambulance clinicians, such initiatives are less likely to succeed.

This study explored professional views about factors affecting OST for suspected stroke patients, with a view to identifying opportunities for interventions designed to reduce OST.

## Methods

### Study design

The study used a generic qualitative research approach (Cooper & Endacott, 2007; Griffiths & Mooney, 2012) involving individual semi-structured interviews with ambulance clinicians. This article is structured using the consolidated criteria for reporting qualitative research (COREQ) checklist (Tong et al., 2007).

### Recruitment

The setting was NEAS, which serves a population of ~2.6 million across urban and rural settings (Office for National Statistics, 2021). Participants were sampled purposively from a pool of paramedics who had volunteered to be interviewed following completion of a survey collecting data about pre-hospital stroke times and care provided for suspected stroke patients (McClelland et al., 2023). Those interviewed were selected from the pool of volunteers so that the sample represented views from paramedics working across the NEAS region. Consent was obtained in writing and reconfirmed verbally before the interview. The number of participants was based on practical considerations pertaining to researcher workload and the study timeline. Complete data saturation was not formally intended (Braun & Clarke, 2021). Participants received a £10 Amazon voucher after their interview.

### Data collection

An interview topic guide (Supplementary 1) was developed by the study team to reflect the study aims and relevant literature; questions were designed to prompt participants to describe how they spent time on scene with stroke patients, explain why they choose to take certain actions on scene with stroke patients, and share their opinions on reasons behind the increase in stroke OST. Questions were adapted as data were collected to enquire about issues highlighted in previous interviews. Interviews were conducted remotely using telephone, Zoom or Microsoft Teams. Interviews were conducted by two researchers based at Newcastle University Stroke Research Group: lead researcher GM, a research paramedic, and AA, a research assistant. Interviews were audio recorded, transcribed verbatim and anonymised. Key points from each interview were summarised and sent back to participants to establish that all key points had been captured. Participants were happy with the summaries and no changes were requested.

### Analysis

Data were transcribed and analysed using thematic analysis (Braun & Clarke, 2021), supported by Quirkos (version 1.6) qualitative analysis software. To ensure credibility of the analysis, data familiarisation, initial coding and development of a provisional thematic structure were first conducted independently by two researchers (GM and AA). Provisional themes were then discussed between researchers to cross-check the analysis and establish a final thematic structure.

## Results

Thirteen paramedics (mean experience of six years, range 3 months to 31 years) took part in the study. Five participants were newly qualified paramedics (NQPs). Three participants were known to GM through prior clinical work. Interviews took place between August and November 2021 and lasted between 33 and 66 minutes. Five interlinked themes were identified from the data, providing insight into factors influencing OST: initial assessment and sources of information; suitability for treatment and interventions; the environment; hospital interactions; and changing practice.

### Theme 1: initial assessment and sources of information

This theme describes how paramedics used OST to gain an understanding of a patient’s condition through assessment, pre-arrival information from ambulance control and other sources such as family members or bystanders.

When arriving on scene, all participants described carrying out an initial rapid ‘eyeball’ review of the patient’s condition, followed by checking vital signs (airway, breathing and circulation) and the face, arms, speech test (FAST). Many participants stated assessments would occur in parallel with taking a brief history from people at scene in efforts to limit OST.

Some participants described carrying out additional advanced neurological assessments as part of their own standard practice. This was usually motivated by the belief that FAST is not comprehensive enough to capture all stroke symptoms.

That’s probably the third patient where I’ve been like, hmm, there’s definitely something going on that the FAST test would not have picked up that needs to be acted upon. I kind of thought why are we as paramedics doing a neurological test as standard that is advertised on television to the public? And you know, we need to be doing a more advanced one. (P5)

Other participants reported that they would only carry out advanced assessments for clarification when they suspected the patient’s symptoms may be caused by a condition other than stroke (i.e. stroke mimic). Another key reason for carrying out advanced assessments on scene was to make better use of time when there were unavoidable delays in extrication, for example during the time on the phone to the stroke ward, or while waiting for back-up.

I had requested some red backup for a crew to take him up to the hospital, we also got some IV access in, and then I did a more advanced neuro assessment. (P5)

Examples of advanced assessments used by participants included the recognition of stroke in the emergency room (ROSIER) scale (Nor et al., 2005), Miami emergency neurologic deficit (MEND) exam (Brotons et al., 2018) and informal assessments guided by individual paramedic experience.

I say, raise your eyebrows, stick your tongue out, puff your cheeks, raise your legs, do this, do that. They think I’m trying to make them do silly things for no reason, and I’m like no, no I’m doing it obviously for a good reason. (P9)

### Theme 2: suitability for treatment and interventions

This theme describes how OST was influenced when paramedics weighed up the potential of individual patients to be eligible for hospital treatment according to emergency care protocols and pre-hospital stroke intervention options.

Many participants reported that symptom onset time was the most salient factor informing their decision making. Specifically, where FAST-positive patients were believed to be within the treatment window for thrombolysis, limiting OST was prioritised.

So, if they’re within four hours, then I move quickly. If they’re outside of the onset, by like, a while, I do tend to move a little bit more slowly. (P1)

The ones where it has just happened are the easy ones – ‘it’s just happened, right let’s go’. (P2)

In these cases, many participants reported only carrying out essential interventions before moving the patient to the vehicle.

Although some participants reported not moving as quickly when they established that the patient was well outside of the treatment window, most participants thought the best course of action for all suspected stroke patients regardless of onset time was to move quickly on scene to get patients to an appropriate point of care.

You might be outside the thrombolysis window, but that doesn’t mean that you can’t be assessed and do something about it. (P4)

Although all participants demonstrated an understanding that limiting OST for stroke patients was important and that meaningful pre-hospital interventions for stroke patients are limited, on-scene actions varied between participants. While the majority believed 12-lead electrocardiograms (ECGs) and cannulation to be unnecessary when treating suspected stroke patients, some reported conducting both as part of their standard practice.

[I always do a] 12-lead ECG. Just to rule everything’s correct as well, because there could be a new atrial fibrillation. (P12)

However, these participants often described carrying out 12-lead ECGs and cannulation in parallel with other activities to minimise OST.

If I am having to cannulate, I would try and just work it around oh well how long is my clinical care assistant going to be getting the bed, getting the chair, what’s it like for access, erm, and try and do something while something else is already being sorted. (P11)

### Theme 3: the environment

This theme describes paramedic experiences and opinions regarding the influence of incident location on OST. This included the immediate location such as the room/floor of a building, and larger geographical considerations such as the nearest hospital and the steps needed to transport the patient to appropriate care.

From a paramedic perspective, once I’ve got the assessment, I can go you know, stable, unstable, obviously it’s an emergency either way, [but I have to consider] how rapidly in terms of how I am going to transport by DCA [double-crewed ambulance] on blue lights, or am I going to try and get an aircraft to fly then if I can, again, depending on where I’m at. It’s a bit different for me now; working up here is on average an hour away from an ED [emergency department]. (P4)

Several participants suggested they often attended suspected stroke patients in care homes and perceived that this may contribute to the increase in OST. Participants described that accessing these patients often requires passing through several locked doors, and that patients are frequently not on the ground floor.

So, sometimes, if you go to a care home, they are always on the upper floor, because they always like to put the most unwell patients on the top floor, as you would, so you’ve got to get through and rely on staff to put the pins in to let you through doors, they’ve usually got to get information for you as well and get charts and stuff. (P1)

Gaining an accurate history about the patient was described as more challenging in care homes, possibly due to regular changeover of staff and many patients lacking capacity. Collectively, these factors were perceived as potential reasons for increased OST.

Several participants said that geographical location influenced actions taken on scene – for instance, with rural patients with a long journey to hospital, they would aim to do minimal observations on scene, choosing to carry out the rest while travelling to save time.

I don’t think this is really a clinical necessity to be doing a 12-lead, but it’s more of a just making sure that I’m not missing something en route. Because I think well, where I’m at up here if I’m transporting anyway me doing a lead on the back of DCA that’s moving and I’ve got an hour to do it, I’m probably more likely to do anyway just for monitoring. (P4)

### Theme 4: hospital interactions

This theme summarises how interactions with hospital staff, both remotely and in person, influenced paramedic actions and OST.

The majority of participants described challenging interactions with stroke unit staff underpinned by what participants described as varying expectations with respect to the information that should be collected on scene.

They [stroke ward staff] are quite terrifying to speak to at times because they, you know they want . . . we all share a common goal of we want the right patients to go to the right place as quickly as possible. But sometimes they can, I think there’s probably a lack of communication in that we don’t know what information they’re looking for until they ask for it. (P5)

Participants described the impact of these interactions on their own practice; some described carrying out additional actions on scene in anticipation of being able to respond to stroke clinician requests.

I think there’s sometimes a blanket cover of, oh well, I’ll do it just because and then I’ll tick the box and then if somebody does come and say oh well, I’ve done it. (P7)

The majority of participants also believed that time spent on the phone to the stroke ward was a key reason for an increase in OST. Several participants contrasted this to old stroke care pathways, in which a phone call to the stroke ward was not required.

You know, it’s not always your two-minute chat, you know, chatting with the nurse and the nurse will say, hang on a minute I’ll put you on the phone with the consultant, they’ll go and find the consultant, then they’ll come back to the phone, ask a few more questions . . . these phone calls are lasting longer and longer . . . I don’t know if that’s the issue as well because again, 10 years ago we didn’t contact the [hospital], we didn’t contact anyone else, we just took everybody to A&E. (P3)

The stroke ward calls take the longest time ever. It can be really quite frustrating, especially when you’ve got somebody that’s coming to the end of the 4-hour onset as well, because you do just want to get them in. But they usually take forever because it’ll be the phone ringing out, then even if they do answer straight away it’ll be the healthcare assistant, and they’ll be like, ‘Oh I’ll just put you on the stroke nurse’, then they’ll ask you a few questions and they’ll say, ‘Oh I’ll just go and speak to the consultant’, then they’ll go and speak to the consultant, they’ll ask them more questions, come back and by the time you’ve actually got through you’ve been like 15 minutes on the phone. (P1)

Several participants felt that using a standardised admission checklist would make telephone contact with the stroke ward more efficient, standardise paramedic actions on scene and reduce OST.

I think it would just be easier if the stroke ward had a standard criteria for what they would accept on the ward. And give that to the ambulance service, as a paper format, and then we can go through it all, put it on the EPCR, we can go through it, ‘do they have this, do they have that, do they have the other’ and then we just ring the stroke ward and say, ‘they have this score, we’re bringing them to the ward’. (P1)

### Theme 5: changing practice

This theme describes how participant experience led to changes in practice regarding management of stroke patients, and participant views on how individual differences in practice may impact on OST. Some participants described the influence of formal education and continuing professional development (CPD) on their practice.

It was an online course, it talked about sort of the different levels of stroke assessment, the basic one being the FAST test and then the ROSIER that they’re using in the majority of hospitals with some variations, and this one [used on scene] was the MEND . . . the Miami emergency neurologic deficit test scale. (P5)

However, the majority reported that their care and management of stroke patients had predominantly been guided by their own experiences and interactions with other healthcare professionals.

I think those questions coming back at you from the stroke nurse are probably prompting you for the next stroke to ask that question next time around. And that might build up to you know, 10 or 20 questions in your head that you want to ask that you’ve been asked. (P8)

So it was actually one of the stroke nurses at the [local hospital] who showed me a technique of doing it [assessing vision]. (P3)

I learned my cranial nerve assessment with an advanced paramedic when I was on placement by accident in [hospital]. (P4)

Many participants felt that the formal training they had received in stroke care was rudimentary and that this made it inadequate for practice.

Several participants perceived that the increase in OST may be due to an increase in on-scene actions carried out by NQPs. Participants often explained this with reference to the influence of university training and NQPs’ motivation to ‘cover all bases’ rather than being guided by practical experience of what works best when managing stroke patients.

What I’ve noticed with NQPs, I don’t know if it’s the way they are taught by the university, is that they do a lot more assessments, a lot more in-depth assessments with patients, on scene. More so maybe than what I do. That in some circumstances may be a downfall on my part, but sometimes on your more time-critical patients, they are still doing the full in-depth assessment, rather than making an early decision, and being confident in making that early decision, without doing everything else, and then moving the patient. I find that this is an issue that I’ve noticed, especially when I’m with NQPs. (P3)

## Discussion

Minimising pre-hospital times through rapid identification and transport is a vital component to improving stroke patient outcomes (Rudd et al., 2020). The need to address increasing ambulance delays in the United Kingdom has been recognised (NHS England, 2022; SSNAP, 2020), however there is little known about paramedic views on factors impacting pre-hospital times. Our study reports the views of 13 paramedics on factors affecting pre-hospital OST for suspected stroke patients. The five themes identified revealed multiple factors as possible influences on OST, which will be discussed in turn with reference to the wider context.

### Theme 1: initial assessment and sources of information

While FAST is the tool recommended by the National Institute for Health and Care Excellence (NICE, 2022) for identification of suspected stroke, many participants in this study reported making use of other tools, such as ROSIER or MEND, typically motivated by the view that FAST is not comprehensive enough to capture all stroke symptoms. While it is correct that FAST cannot identify all stroke presentations (e.g. those with sudden onset visual disturbance or lateralising cerebellar dysfunction; NICE, 2022), it quickly recognises most stroke patients, with a sensitivity of 79–87% (Powers et al., 2019). Additionally, a previous evaluation comparing FAST and ROSIER by the London Ambulance Service concluded that ROSIER was not superior to FAST in identification of stroke patients (Fothergill et al., 2013). Paramedic training should emphasise that in straightforward acute presentations, FAST-positive patients should be treated as suspected stroke, and additional assessment should be reserved for when there is uncertainty about whether symptoms could indicate a different problem.

### Theme 2: suitability for treatment and interventions

While most participants reported trying to minimise additional patient care actions for suspected stroke patients, this was not ubiquitous across the sample, and has previously been recognised as a reason for delays. A study from Denmark (Drenck et al., 2019) described factors such as communication difficulties, vomiting, recording ECGs and placing intravenous (IV) cannulas as extending OST. A US paper (Li et al., 2021) also described how 12-lead ECG, blood glucose measurement and IV cannulation contribute to OST, while systematic review data (Munro et al., 2018) describe the association between pre-hospital 12-lead ECGs, increased OST and worse patient outcomes. Paramedic training should therefore focus on raising awareness that meaningful on-scene interventions for stroke patients are limited, and that actions such as 12-lead ECGs and IV cannulation are likely to increase OST and be of detriment to patient outcomes.

### Theme 3: the environment

In the environmental factors theme, participants described factors that are largely unmodifiable, such as patient location and distance to hospital. The perception that attending suspected stroke patients in care homes extends OST is beyond the control of the attending paramedic but may suggest that further training is needed in care homes to promote efficient access and communication when there is emergency attendance. Participants described adapting their practice depending on the nearest hospital, for example carrying out only essential on-scene actions for patients where conveyance to hospital would take longer and carrying out additional interventions (e.g. IV cannulation) to meet the preference of an admitting hospital. This is an understandable reaction to locality-specific stroke pathways (McClelland et al., 2018), but introduces variability which is not ideal for minimising delays. The recent NHS England Getting It Right First Time report highlighted the importance of reducing variability in stroke care to improve patient outcomes (Getting It Right First Time, 2022). Therefore, interventions designed to optimise pre-hospital stroke care might focus on standardising actions taken by paramedics when on scene with stroke patients.

### Theme 4: hospital interactions

Pre-alerting stroke patients improves time to interventions in hospital such as thrombolysis (Hsieh et al., 2016), however previous studies have indicated that the method of communicating the pre-alert may impact OST. Gunn (2021) reported that pre-alerts given via radio were quicker than those given by telephone. Consistent with this finding, participants in this study perceived that time spent on the phone to the stroke ward was a key contributor to OST, but indicated that it was not telephone as a method of pre-alert per se causing the increase in OST but the nature of interactions with stroke units. Paramedics reported that the format of phone calls was frequently variable and inefficient, and that they could not always anticipate the information stroke units would request so carried out additional actions on scene to feel prepared for exchanges. Several papers report the potential benefit of standardised communication processes between pre-hospital and hospital staff (Flynn et al., 2017; Loseby et al., 2013; Wood et al., 2015). As such, it may be that interventions to establish standard pre-hospital assessment and communication processes may be of similar benefit to OST.

### Theme 5: changing practice

Previous research in pre-hospital stroke care has shown that around one-third of paramedics rated their initial stroke training as inadequate, and that many feel additional stroke training would be beneficial (McClelland et al., 2017). These findings are mirrored by this study; most participants felt that their training relating to managing stroke patients was rudimentary, with many reporting that most of their methods were guided by their own individual experience and information acquired from informal sources (i.e. other clinicians ‘on the job’), rather than from prescribed stroke education or CPD. As such, though the specific impact of stroke CPD on OST has not been explored, interventions to target OST may consider improving access to CPD on stroke for paramedics. Indeed, it is reasonable to assume that further training to enhance paramedics’ pre-hospital management of stroke patients may help to optimise their choice of on-scene actions, which could in turn reduce OST.

Several participants suggested that NQPs carry out more actions on scene, which may contribute to extended OST. Previous literature exploring Canadian paramedic perspectives on trauma care suggests that greater experience may lead to better OST management (Levitan et al., 2018). Similarly, Robinson (2017) highlights literature which demonstrates a relationship between experience and clinical decision making, resource management and rapid treatment, while Holmes et al. (2017) indicates that student paramedics are generally less confident in their clinical decision making, which may lead them to spend extra time on scene. NQP education may also have an impact on stroke OST, however the nature of NQP education varies widely depending on the training route taken or university attended, and the relationship between NQP education and stroke OST is yet to be explored. Collectively, as there is no objective evidence to suggest a specific link between NQP education, clinical experience and OST, further research is needed to decipher whether these factors have an independent influence on OST.

Collectively, this study signposts several potential targets for interventions to reduce OST, as well as numerous avenues for further OST research. Standardising on-scene assessments for stroke patients, refining communication processes between ambulance services and hospital stroke services and increasing availability of stroke CPD for paramedics may all serve to reduce OST, while further research exploring the relationship between clinical experience, NQP education and CPD on OST may help to elucidate other potential factors influencing stroke OST.

### Limitations

Limitations include the involvement of a small paramedic-only sample from a single ambulance service covering one region of England, which may mean that findings are not representative of views from the whole service. GM knew some of the participants and the nature of his work may have been known to participants, which could have influenced their willingness to speak openly. This study was also conducted during a period of intense pressure on the ambulance service during the second year of the COVID-19 pandemic, which may have influenced participants’ views.

## Conclusion

This study provides insight into how UK ambulance clinicians spend time on scene with suspected stroke patients. Multiple factors influencing OST were identified. Several of the factors identified are potentially modifiable and may act as suitable opportunities for interventions designed to reduce OST and for future pre-hospital research. The findings of this study may also be used to inform development of the most efficient care pathway for suspected stroke patients.

## Acknowledgements

The authors would like to thank the paramedics who agreed to be interviewed for their support with this project.

## Author contributions

All authors were involved in the development of this study. GM and AA conducted the interviews. All authors were involved in the analysis of the data. AA drafted the paper, which was reviewed and approved by all authors. AA acts as the guarantor for this article.

## Conflict of interest

GM is the editor-in-chief of the *BPJ*.

## Ethics

Approvals were secured from the Health Research Authority (Ref 295751), Newcastle University ethics committee (Ref 9631/2020) and NEAS as the sponsor. Research Ethics Committee approval was not necessary as this was a staff-focused study. The study was adopted onto the NHS CRN portfolio. Participants who volunteered for the study were provided with a participant information sheet, were given the opportunity to ask questions and gave written consent prior to the interviews, which was reconfirmed verbally at the time of the interview. Participants were told they could withdraw from the study at any time without providing explanation.

## Funding

This project was funded by a Stroke Association post-doctoral fellowship awarded to GM (ref SA PDF 20\100001).

## References

[bibr_1] Braun V., & Clarke V. (2021). *Thematic analysis: A practical guide to understanding and doing* (1st ed.). SAGE Publications.

[bibr_2] Brotons A. A. Motola I. Rivera H. F. Soto R. E. Schwemmer S., & Issenburg S. B. (2018). Correlation of the Miami emergency neurologic deficit (MEND) exam performed in the field by paramedics with an abnormal NIHSS and final diagnosis of stroke for patients airlifted from the scene. *Stroke*, 43, A3468. https://doi.org/10.1161/str.43.suppl_1.A3468.

[bibr_3] Cooper S., & Endacott R. (2007). Generic qualitative research: A design for qualitative research in emergency care? *Emergency Medicine Journal*, 24(12), 816–819. 18029510 10.1136/emj.2007.050641 PMC2658349

[bibr_4] Drenck N. Viereck S. Baekgaard J. S. Christensen K. B. Lippert F., & Folke F. (2019). Pre-hospital management of acute stroke patients eligible for thrombolysis – an evaluation of ambulance on-scene time. *Scandinavian Journal of Trauma Resuscitation & Emergency Medicine*, 27(1), 3. https://doi.org/10.1186/s13049-018-0580-4. 30626404 10.1186/s13049-018-0580-4 PMC6327613

[bibr_5] Evans M. R. White P. Cowley P., & Werring D. J. (2017). Revolution in acute ishcheamic stroke care: A practical guide to mechanical thrombectomy. *Practical Neurology*, 17(4), 252–265. 28647705 10.1136/practneurol-2017-001685 PMC5537551

[bibr_6] Flynn D. Francis R. Robalino S. Lally J. Snooks H. Rodgers H. McClelland G. Ford G. A., & Price C. (2017). A review of enhanced paramedic roles during and after hospital handover of stroke, myocardial infarction and trauma patients. *BMC Emergency Medicine*, 17(1), 5. https://doi.org/10.1186/s12873-017-0118-5. 28228127 10.1186/s12873-017-0118-5 PMC5322648

[bibr_7] Fothergill R. T. Williams J. Edwards M. J. Russell I. T., & Gompertz P. (2013). Does use of the recognition of stroke in the emergency room stroke assessment tool enhance stroke recognition by ambulance clinicians? *Stroke*, 44(11), 3007–3012. 24072006 10.1161/STROKEAHA.13.000851

[bibr_8] Fouche P. F. Smith K. Jennings P. A. Boyle M., & Bernard S. (2019). The association of paramedic rapid sequence intubation and survival in out-of-hospital stroke. *Emergency Medicine Journal*, 36(7), 416–422. 31147349 10.1136/emermed-2019-208613

[bibr_9] Fulop N. J. Ramsey A. I. G. Hunter R. M. McKevitt C. Perry C. Turner S. J. Boaden R. Papachristou I. Rudd A. G. Tyrell P. J. Wolfe C. D. A., & Morris S. (2019). Evaluation of reconfigurations of acute stroke services in different regions of England and lessons for implementation: A mixed-methods study. *Health and Social Care Delivery Research*, 7(7). https://doi.org/10.3310/hsdr07070. 30789689

[bibr_10] Getting It Right First Time. (2022). *Stroke: GIRFT programme national speciality report*. https://gettingitrightfirsttime.co.uk/girft-reports/.

[bibr_11] Griffiths P., & Mooney G. P. (2012). *The paramedic’s guide to research: An introduction*. McGraw-Hill/Open University Press.

[bibr_12] Gunn J. (2021). Do methods of hospital pre-alerts influence the on-scene times for acute pre-hospital stroke patients? A retrospective observational study. *British Paramedic Journal*, 6(2), 19–25. 34539251 10.29045/14784726.2021.9.6.2.19 PMC8415206

[bibr_13] Haworth D., & McClelland G. (2019). Call to hospital times for suspected stroke patients in the North East of England: A service evaluation. *British Paramedic Journal*, 4(2), 31–36. 10.29045/14784726.2019.09.4.2.31 PMC7706759 33328834

[bibr_14] Holmes L. Jones R. Brightwell R., & Cohen L. (2017). Student paramedic anticipation, confidence and fears: Do undergraduate courses prepare student paramedics for the mental health challenges of the profession? *Australasian Journal of Paramedicine*, 14(4). https://doi.org/10.33151/ajp.14.4.545.

[bibr_15] Hsieh M.-J. Tang S.-C. Chiang W.-C. Tsai L.-K. Jeng J.-S. Ma M. H.-M., & Taipei EMS Stroke Collaborative Group. (2016). Effect of prehospital notification on acute stroke care: A multicenter study. *Scandinavian Journal of Trauma Resuscitation & Emergency Medicine*, 24, 57. https://doi.org/10.1186/s13049-016-0251-2. 27121501 10.1186/s13049-016-0251-2 PMC4847216

[bibr_16] Langhorne P. Audebert H. J. Cadilhac D. A. Kim J., & Lindsay P. (2020). Stroke systems of care in high-income countries: What is optimal? *Lancet*, 396(10260), 1433–1442. 33129394 10.1016/S0140-6736(20)31363-5

[bibr_17] Levitan M. Law M. P. Ferron R., & Lutz-Graul K. (2018). Paramedics’ perspectives on factors impacting on-scene times for trauma calls. *Prehospital and Disaster Medicine*, 33(3), 250–255. 29729684 10.1017/S1049023X18000389

[bibr_18] Li T. Cushman J. T. Shah M. N. Kelly A. G. Rich D. Q., & Jones C. M. C. (2021). Prehospital time intervals and management of ischemic stroke patients. *American Journal of Emergency Medicine*, 42, 127–131. 32059935 10.1016/j.ajem.2020.02.006

[bibr_19] London Ambulance Service NHS Trust. (2018). *Stroke annual report 2017–18*. https://www.londonambulance.nhs.uk/?dlm_download_category=stroke-reports.

[bibr_20] Loseby J. Hudson A., & Lyon R. (2013). Clinical handover of the trauma and medical patient: A structured approach. *Journal of Paramedic Practice*, 5(10), 563–567.

[bibr_21] McClelland G. Burrow E. Alton A. Shaw L. Finch T., & Price C. (2023). What factors contribute towards ambulance on-scene times for suspected stroke patients? An observational study. *European Stroke Journal* 8(2), 492–500. 37231700 10.1177/23969873231163290 PMC10334177

[bibr_22] McClelland G. Flynn D. Rodgers H., & Price C. (2017). A survey of UK paramedics’ views about their stroke training, current practice and the identification of stroke mimics. *British Paramedic Journal*, 2(1), 4–15.

[bibr_23] McClelland G. Rodgers H., & Price C. (2018). A survey of pre-hospital stroke pathways used by UK ambulance services. *International Journal of Stroke*, 13(3_suppl), 10–65.

[bibr_24] Munro S. F. S. Cooke D. Kiln-Barfoot V., & Quinn T. (2018). The use and impact of 12-lead electrocardiograms in acute stroke patients: A systematic review. *European Heart Journal-Acute Cardiovascular Care*, 7(3), 257–263. 26637212 10.1177/2048872615620893

[bibr_25] NHS England. (2022). *NHS England’s work on stroke*. https://www.england.nhs.uk/ourwork/clinical-policy/stroke/.

[bibr_26] NICE. (2022). *Stroke and transient ischaemic attack*. https://www.nice.org.uk/guidance/conditions-and-diseases/cardiovascular-conditions/stroke-and-transient-ischaemic-attack.

[bibr_27] Nor A. M. Davis J. Sen B. Shipsey D. Louw S. J. Dyker A. G. Davis M., & Ford G. A. (2005). The recognition of stroke in the emergency room (ROSIER) scale: Development and validation of a stroke recognition instrument. *Lancet Neurology*, 4(11), 727–734. 16239179 10.1016/S1474-4422(05)70201-5

[bibr_28] Office for National Statistics. (2021). *Population and household estimates, England and Wales: Census 2021*. https://www.ons.gov.uk/peoplepopulationandcommunity/populationandmigration/populationestimates/datasets/populationandhouseholdestimatesenglandandwalescensus2021.

[bibr_29] Powers W. Rabinstein A. A. Ackerson T. Adeoye O. M. Bambakidis N. C. Becker K. Biller J. Brown M. Demaerschalk B. M. Hoh B. Jauch E. C. Kidwell C. S. Leslie-Mazwi T. M. Ovbiagele B. Scott P. A. Sheth K. N. Southerland A. M. Summers D. V. Tirschwell D. L., & and on behalf of the American Heart Association Stroke Council. (2019). Guidelines for the early management of patients with acute ischemic stroke: 2019 update to the 2018 guidelines for the early management of acute ischemic stroke: A guideline for healthcare professionals from the American Heart Association/American Stroke Association. *Stroke*, 50(12), e344–e418. 31662037 10.1161/STR.0000000000000211

[bibr_30] Price C. I. Rae V. Duckett J. Wood R. Gray J. McMeekin P. Rodgers H. Portas K., & Ford G. A. (2013). An observational study of patient characteristics associated with the mode of admission to acute stroke services in North East, England. *PLoS One*, 8(10), e76997. https://doi.org/10.1371/journal.pone.0076997. 24116195 10.1371/journal.pone.0076997 PMC3792886

[bibr_31] Puolakka T. Kuisma M. Lankimaki S. Puolakka J. Hallikainen J. Rantanen K., & Lindsberg P. J. (2016). Cutting the prehospital on-scene time of stroke thrombolysis in Helsinki: A prospective interventional study. *Stroke*, 47(12), 3038–3040. 27827326 10.1161/STROKEAHA.116.014531

[bibr_32] Robinson S. (2017). A student perspective: Newly qualified paramedic programme. *Journal of Paramedic Practice*, 9(6), 238–239.

[bibr_33] Rudd A. G. Bladin C. Carli P. De Silva D. A. Field T. S. Jauch E. C. Kudenchuk P. Kurz M. W. Laerdal T. Ong M. E. H. Panagos P. Ranta A. Rutan C. Sayre M. R. Schonau L. Shin S. D. Waters D. Lippert F., & Grp U. S. W. (2020). Utstein recommendation for emergency stroke care. *International Journal of Stroke*, 15(5), 555–564. 32223543 10.1177/1747493020915135 PMC7672780

[bibr_34] Schwartz J. Dreyer R. P. Murugiah K., & Ranasinghe I. (2016). Contemporary prehospital emergency medical services response times for suspected stroke in the United States. *Prehospital Emergency Care*, 20(5), 560–565. 26953776 10.3109/10903127.2016.1139219

[bibr_35] Simonsen S. A. Andresen M. Michelsen L. Viereck S. Lippert F. K., & Iversen H. K. (2014). Evaluation of pre-hospital transport time of stroke patients to thrombolytic treatment. *Scandinavian Journal of Trauma Resuscitation & Emergency Medicine*, 22, 65. https://doi.org/10.1186/s13049-014-0065-z. 25391354 10.1186/s13049-014-0065-z PMC4233232

[bibr_36] SSNAP. (2020). *The seventh annual SSNAP report*. https://www.strokeaudit.org/Quality-Improvement/Appraisal-and-Revalidation-(1).aspx.

[bibr_37] Tong A. Sainsbury P., & Craig J. (2007). Consolidated criteria for reporting qualitative research (COREQ): A 32-item checklist for interviews and focus groups. *International Journal for Quality in Health Care*, 19(6), 349–357. 17872937 10.1093/intqhc/mzm042

[bibr_38] Wardlaw J. M. Murray V. Berge E., & del Zoppo G. J. (2014). Thrombolysis for acute ischemic stroke, update August 2014. *Stroke*, 45(11), e222–e225.

[bibr_39] Wood K. Crouch R. Rowland E., & Pope C. (2015). Clinical handovers between prehospital and hospital staff: Literature review. *Emergency Medicine Journal*, 32(7), 577–581. 25178977 10.1136/emermed-2013-203165

